# Elucidating the role of TWIST1 in ulcerative colitis: a comprehensive bioinformatics and machine learning approach

**DOI:** 10.3389/fgene.2024.1296570

**Published:** 2024-03-06

**Authors:** Wenjie Ou, Zhaoxue Qi, Ning Liu, Junzi Zhang, Xuguang Mi, Yuan Song, Yanqiu Fang, Baiying Cui, Junjie Hou, Zhixin Yuan

**Affiliations:** ^1^ School of Clinical Medicine, Changchun University of Chinese Medicine, Changchun, Jilin, China; ^2^ Department of Secretory Metabolism, The First Hospital of Jilin University, Changchun, Jilin, China; ^3^ General Surgery of The First Clinical Hospital of Jilin Academy of Chinese Medicine Sciences, Changchun, Jilin, China; ^4^ Department of Central Laboratory, Jilin Provincial People’s Hospital, Changchun, Jilin, China; ^5^ Department of Gastroenterology, Jilin Provincial People’s Hospital, Changchun, Jilin, China; ^6^ Department of Comprehensive Oncology, Jilin Provincial People’s Hospital, Changchun, Jilin, China; ^7^ Department of Emergency Surgery, Jilin Provincial People’s Hospital, Changchun, Jilin, China

**Keywords:** ulcerative colitis (UC), Twist1, bioinformatics, machine learning, gene expression omnibus (GEO) database, differentially expressed genes (DEGs), diagnostic marker

## Abstract

**Background:** Ulcerative colitis (UC) is a common and progressive inflammatory bowel disease primarily affecting the colon and rectum. Prolonged inflammation can lead to colitis-associated colorectal cancer (CAC). While the exact cause of UC remains unknown, this study aims to investigate the role of the TWIST1 gene in UC.

**Methods:** Second-generation sequencing data from adult UC patients were obtained from the Gene Expression Omnibus (GEO) database. Differentially expressed genes (DEGs) were identified, and characteristic genes were selected using machine learning and Lasso regression. The Receiver Operating Characteristic (ROC) curve assessed TWIST1’s potential as a diagnostic factor (AUC score). Enriched pathways were analyzed, including Gene Ontology (GO), Kyoto Encyclopedia of Genes and Genomes (KEGG), and Gene Set Variation Analysis (GSVA). Functional mechanisms of marker genes were predicted, considering immune cell infiltration and the competing endogenous RNA (ceRNA) network.

**Results:** We found 530 DEGs, with 341 upregulated and 189 downregulated genes. TWIST1 emerged as one of four potential UC biomarkers via machine learning. TWIST1 expression significantly differed in two datasets, GSE193677 and GSE83687, suggesting its diagnostic potential (AUC = 0.717 in GSE193677, AUC = 0.897 in GSE83687). Enrichment analysis indicated DEGs associated with TWIST1 were involved in processes like leukocyte migration, humoral immune response, and cell chemotaxis. Immune cell infiltration analysis revealed higher rates of M0 macrophages and resting NK cells in the high TWIST1 expression group, while TWIST1 expression correlated positively with M2 macrophages and resting NK cell infiltration. We constructed a ceRNA regulatory network involving 1 mRNA, 7 miRNAs, and 32 long non-coding RNAs (lncRNAs) to explore TWIST1’s regulatory mechanism.

**Conclusion:** TWIST1 plays a significant role in UC and has potential as a diagnostic marker. This study sheds light on UC’s molecular mechanisms and underscores TWIST1’s importance in its progression. Further research is needed to validate these findings in diverse populations and investigate TWIST1 as a therapeutic target in UC.

## 1 Introduction

Ulcerative Colitis (UC) is a chronic form of Inflammatory Bowel Disease (IBD) primarily affecting the colon and rectum. The exact cause of UC remains elusive, although several factors, including genetics, environmental triggers, and immune responses, are believed to play pivotal roles in its onset (Kobayashi et al., 2020). While the incidence of adult UC in Asia has historically been relatively low, there has been a noticeable increase in recent years (Du and Ha, 2020). UC not only significantly impairs the quality of life for affected individuals but, in severe cases, also raises the risk of developing colitis-associated colorectal cancer (CAC) (Yashiro, 2014). Hence, the exploration of potential risk markers highly correlated with the occurrence and progression of UC is of paramount importance.

TWIST1, a basic helix-loop-helix (bHLH) transcription factor, was initially identified during embryonic development and plays a pivotal role in cellular migration, differentiation, and morphogenesis (Murre et al., 1989; Jan and January 1993; Kadesch, 1993). In oncological research, the Twist1 gene has garnered significant attention due to its cardinal role in tumor invasion and metastasis (Ren et al., 2016; Ghafouri-Fard et al., 2021). However, the implications of the Twist1 gene in UC remain largely uncharted. A study from June 2018 highlighted that the expression of TWIST1 protein was markedly elevated in tissues from both UC and CAC, and it was closely associated with tissue cellular apoptosis (Anonymous, 2023). Limitations of this study include the exclusive use of immunohistochemistry techniques to investigate gene expression levels within tissues. As a result, it did not explore the correlation between TWIST1 expression and immune factors closely associated with the occurrence and development of UC and CAC. Furthermore, it did not investigate the relationship between TWIST1 expression and the activity of UC. The search for transcriptional regulators of TWIST1 and the exploration of its regulatory targets were also omitted, although these aspects are considered indispensable.

With the advent of bioinformatics and high-throughput sequencing technologies, researchers have pinpointed several genes and pathways intrinsically linked to UC, offering fresh insights into its intricate pathophysiological mechanisms (Kakiuchi et al., 2020; Tong et al., 2021; Xu et al., 2022). Bioinformatics provides a robust analytical framework for identifying pivotal genes associated with UC and analyzing their expression significance. This study harnesses the second-generation sequencing data of adult UC from the Gene Expression Omnibus (GEO) database to probe potential aberrations in the expression levels of the TWIST1 gene. Additionally, the burgeoning field of machine learning bestows capabilities in predictive modeling and pattern discernment, proving indispensable in the interpretation of multifaceted biological datasets. Functional enrichment analysis further facilitates a profound comprehension of the biological intricacies of genes. By juxtaposing differentially expressed genes (DEGs) with functional databases, we endeavor to unveil the biological pathways and processes in which TWIST1 partakes, thereby delving into its prospective role in UC pathogenesis. This research aspires to elucidate the nexus between the TWIST1 gene and UC, fortifying our understanding of its disease mechanisms and informing future therapeutic paradigms.

## 2 Materials and methods

### 2.1 Selection and download of the UC dataset

We retrieved matrix files from the GEO database (https://www.ncbi.nlm.nih.gov/geo/) that contained samples of normal human intestinal mucosal tissue and intestinal mucosal tissue from adult patients with UC. Our selection process followed specific criteria: (1) The data pertained to high-throughput sequencing expression profiles of *Homo sapiens*; (2) The samples included biopsied intestinal mucosal tissue from both healthy adults and UC patients; (3) Samples were taken from patients with active clinical disease; (4) Each dataset contained over 6 samples; (5) All the included samples had not been subjected to drug treatment; (6) The dataset provided comprehensive information about each sample. As a result, we identified two datasets for our study. The first, GSE193677 (Argmann et al., 2023), encompassed a total of 461 samples from healthy human subjects (control group) and 126 samples from patients with UC (treatment group). Furthermore, for subsequent validation, we opted for the GSE83687 (Peters et al., 2017) datasets, consisting of 60 samples from healthy human colon tissue and 32 samples from UC-affected colon tissue, as depicted in Table 1. It is worth noting that data from the GEO database is readily accessible to the public, obviating the need for local ethics committee approval.

**TABLE 1:** Information for selected microarray datasets.

GEO accession	Samples		Country	Attribute
	Con	UC		
GSE193677	461	126	United States	Test set
GSE83687	60	32	United States	Validation set

### 2.2 Correction, screening and visualization of differentially expressed genes

After downloading the matrix files from the GEO database, we proceeded to process and annotate them utilizing both Perl language (version 32), R language (version 4.30), and Excel. DEGs were derived by subjecting the sample data to filtration through the R limma package. Our filtration criteria encompassed |Log Fold Change (FC)| > 1, and the ensuing *p*-values underwent correction while controlling the false discovery rate (FDR), resulting in an adjusted *p*-value (Q value) < 0.05. Subsequently, the chosen DEGs were subject to visualization and analysis, and the outcome was the generation of heat maps and volcano plots.

### 2.3 Utilizing machine learning for the identification of disease-related genes

We proceeded to employ machine learning techniques for the additional screening of the acquired DEGs, with the objective of pinpointing genes with a high degree of association with UC. TWO distinct machine learning algorithms, namely, the Least Absolute Shrinkage and Selection Operator (LASSO) (Tibshirani, 1996) and the Support Vector Machine with Recursive Feature Elimination (SVM-RFE) (Suykens and Vandewalle, 1999), were employed to effectively sift through the pool of DEGs. Finally, R venn package was use to obtain their intersection genes. This enabled us to pinpoint potential disease biomarkers with remarkable precision.

### 2.4 Validation of TWIST1 expression and diagnostic value

In the GSE193677 and GSE83687 datasets, *t*-test was employed to compare the expression levels of TWIST1 between the UC experimental group and the control group. Sensitivity and specificity of TWIST1 were determined through Receiver Operating Characteristic (ROC) (Kumar and Indrayan, 2011) curve analysis using the R pROC package. These results were visually depicted using the R ggplot2 package.

Furthermore, Clinical information data for the GSE193677 dataset were obtained, and clinical disease activity was categorized as active or inactive. Kruskal–Wallis tests were conducted to assess the association between clinical disease activity and TWIST1 expression levels in both the UC experimental group and the control group. Statistical significance was defined when the *p*-values from both tests were below 0.05.

### 2.5 Difference analysis based on the median value of TWIST1 gene expression

Within GSE193677, division into two distinct groups was undertaken based on the distinct levels of TWIST1 expression. Employing identical methods and parameters outlined earlier, DEGs were filtered within these two groups, categorized as TWIST1 high- and low-expression groups. Analysis of DEGs between these groupings was executed via the R “Limma” package, and differential expression was visualized utilizing the R “ggplot2″ package through the creation of volcano plots (*p*-values below 0.05 and |log2FC| exceeding 1).

### 2.6 Functional enrichment and gene regulatory networks analysis

Utilizing the R “clusterProfiler” package (Yu et al., 2012), Gene Ontology (GO) (Ashburner et al., 2000) analysis was conducted between elevated and diminished TWIST1 expression levels within UC samples to elucidate the implicated biological processes (BP), molecular functions (MF), and cellular components (CC). The identification of signaling pathways linked to TWIST1-associated DEGs was accomplished through Kyoto Encyclopedia of Genes and Genomes (KEGG) (Ogata et al., 1999) pathway analysis. Utilizing the “GSVA” package in R (Hänzelmann et al., 2013), the UC dataset was transformed into a gene set expression matrix. Gene Set Variation Analysis (GSVA) was then employed to meticulously examine the variations in GO and KEGG enrichment between the TWIST1 high-expression and low-expression groups. Notably, to achieve significant enrichment, the |t| value was mandated to exceed 5 for the Hallmark genome.

Adhering to the ceRNA hypothesis, predictions of TWIST1-bound miRNAs were derived using the TargetScan database (https://www.targetscan.org/vert_80/), miRDB database (http://www.mirdb.org/), and the Miranda database (https://cbio.mskcc.org/miRNA2003/miranda. html). Simultaneously, the spongeScan database (https://bioinformaticshome. com/index. html) was employed for the prediction of associated lncRNAs. The resultant networks were subsequently fine-tuned and visually represented using Cytoscape 3.92 software (Otasek et al., 2019).

### 2.7 Immune cell infiltrates and correlation between TWIST1

The quantification of 22 immune cell types within UC samples was accomplished through the utilization of the “CIBERSORT” software package (Newman et al., 2015). For a more in-depth analysis, exclusively data with a CIBERSORT value below *p* < 0.05 were retained. This selective process yielded a matrix detailing the fractions of immune cells present. The evaluation of immune infiltration disparity between the TWIST1 high expression and low expression groups was conducted via the Wilcoxon rank sum test. Employing the “boxplot” function within the R software package, we visually depicted the contrast in immune cell infiltration levels between the two TWIST1 expression groups.

Moreover, we conducted Spearman correlation analysis to investigate the potential linkage between TWIST1 expression and immune cell infiltration. For visualization purposes, the R ggplot2 package was harnessed, allowing us to graphically represent these associations.

## 3 Results

### 3.1 Findings from genes exhibiting differential expression

**FIGURE 1:**
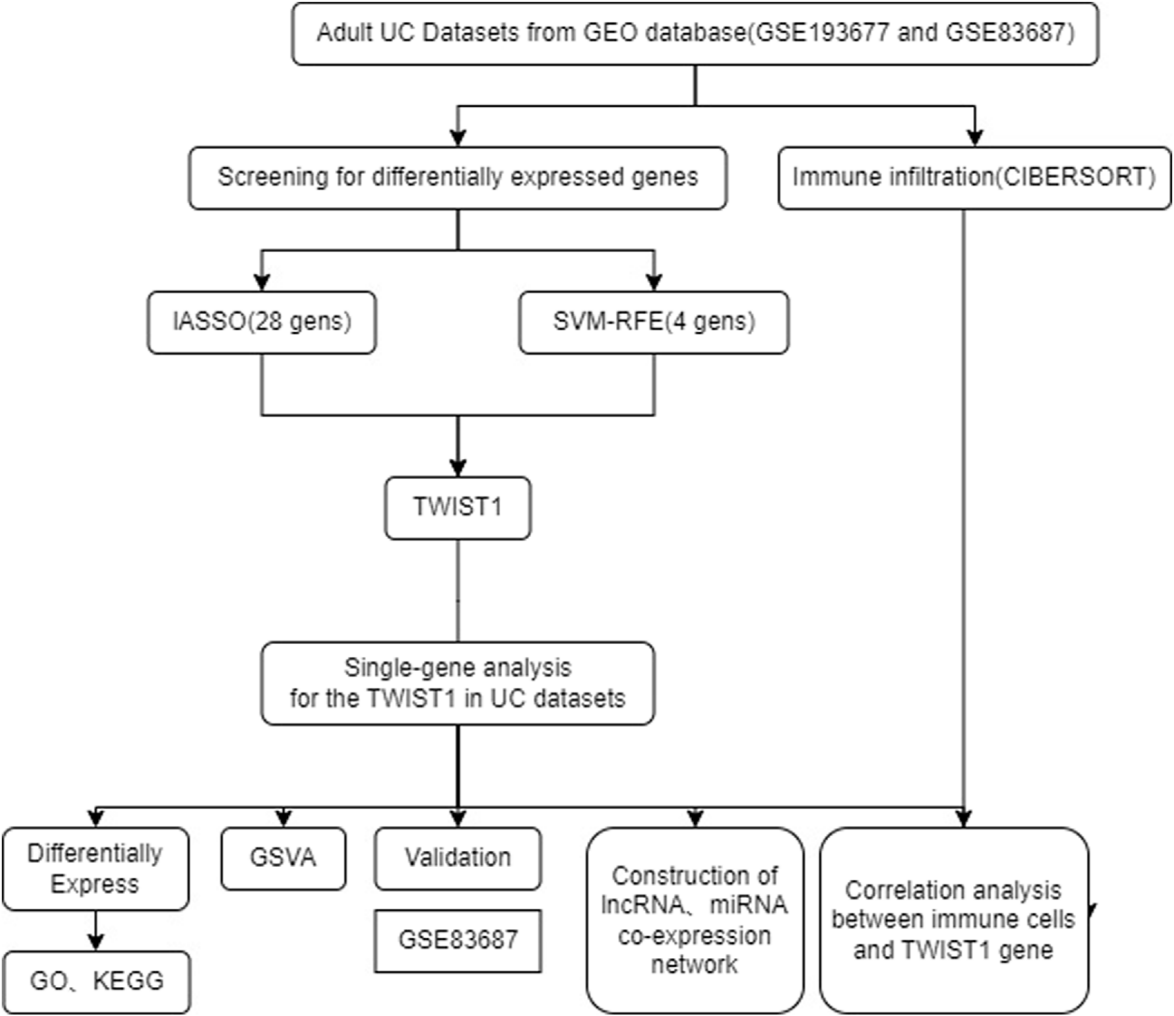
The flowchart of the analysis.

**FIGURE 2:**
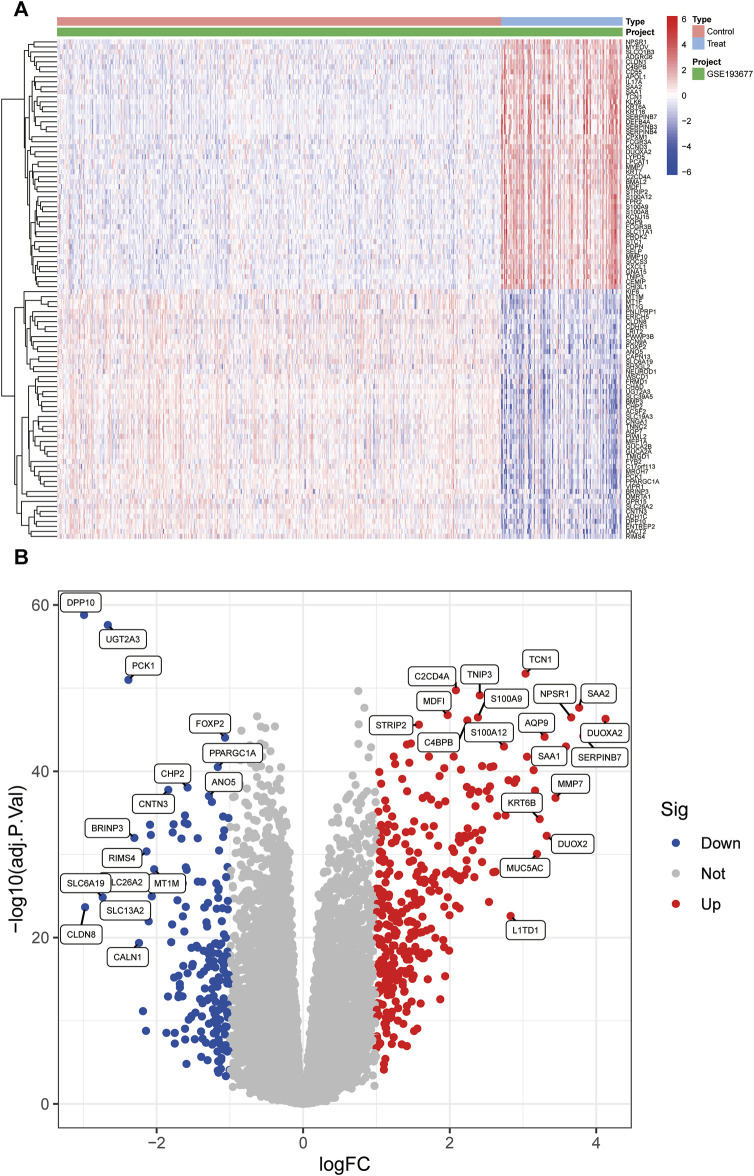
The heatmap and a volcano plots. **(A)** The heatmap of DEGs distribution; **(B)** The volcano plots of DEGs. Red represented a high expression of DEG, while blue represented a low expression of DEG.

The schematic portrayal of our study’s methodology is depicted (Figure 1). Inclusion comprised of 461 samples obtained from healthy human intestinal mucosal biopsies (con group) and 126 samples from patients with active colonic mucosal tissue affected by UC (treat group), all sourced from the GSE193677 datasets. A comprehensive screening yielded a tally of 530 DEGs, encompassing 341 genes exhibiting upregulation and 189 genes manifesting downregulation, as highlighted (Figures 2A, B).

### 3.2 Machine learning to screen potential biomarkers and its diagnostic value

**FIGURE 3:**
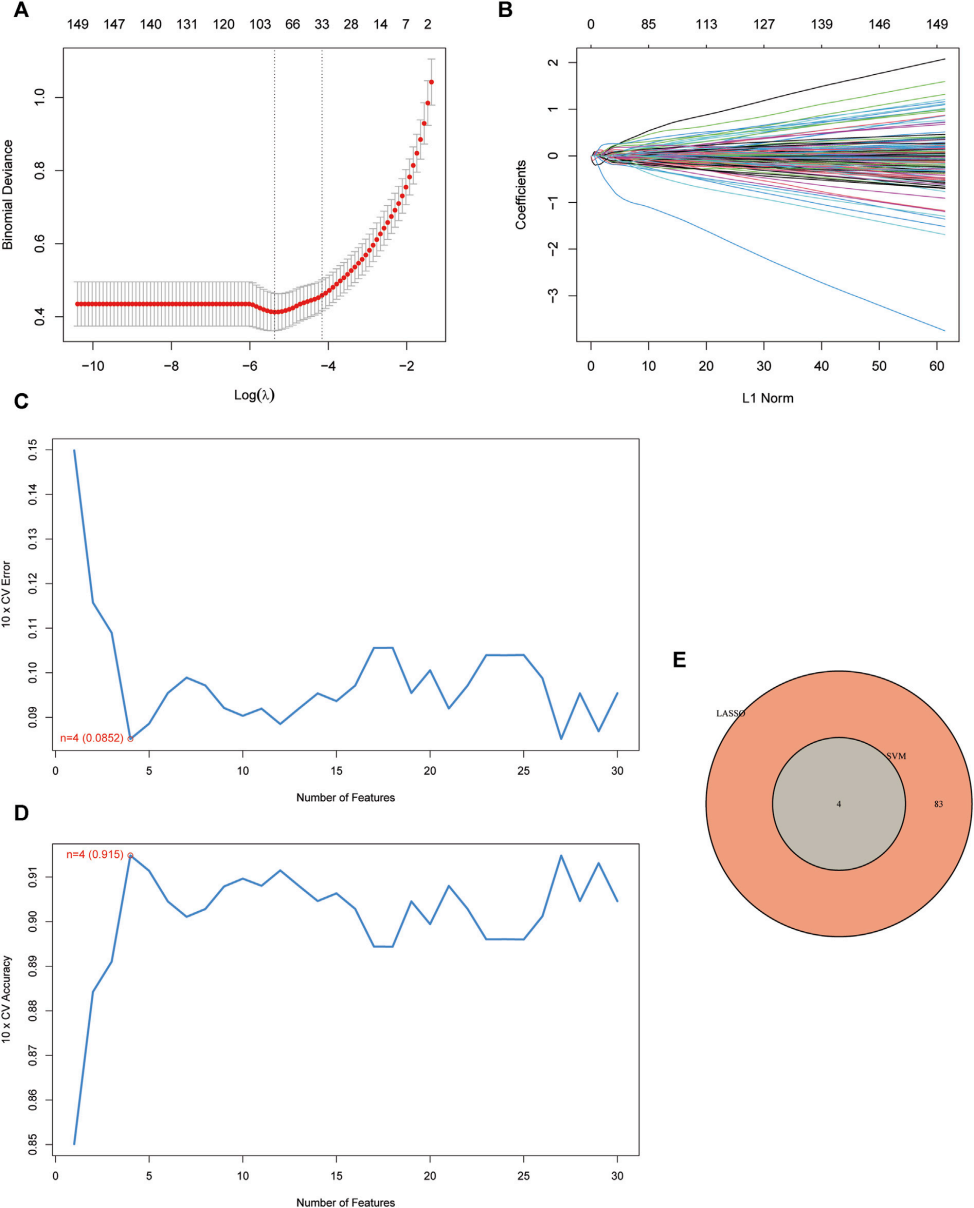
Screening of disease-related genes by machine learning. **(A, B)** Regression coefficient path diagram and cross-validation curves in LASSO logistic regression algorithm.; **(C, D)** The curve of change in the predicted true and error value of each gene in SVM-RFE algorithm.; **(E)** Venn diagram demonstrates the intersection of diagnostic markers obtained from the three algorithms.

The LASSO logistic regression method pinpointed 87 genes as potential UC biomarkers (Figures 3A, B). Subsequently, we selected features and identified 4 optimal UC candidate genes through SVM-RFE (Figures 3C, D). The overlap between the two algorithms yielded a set of 4 genes: S100 Calcium Binding Protein P (S100P), The G protein-coupled receptor 15 (GPR15), Twist Family BHLH Transcription Factor 1 (TWIST1), and Rho Family GTPase 1 (RND1) (Figure 3E).

**FIGURE 4:**
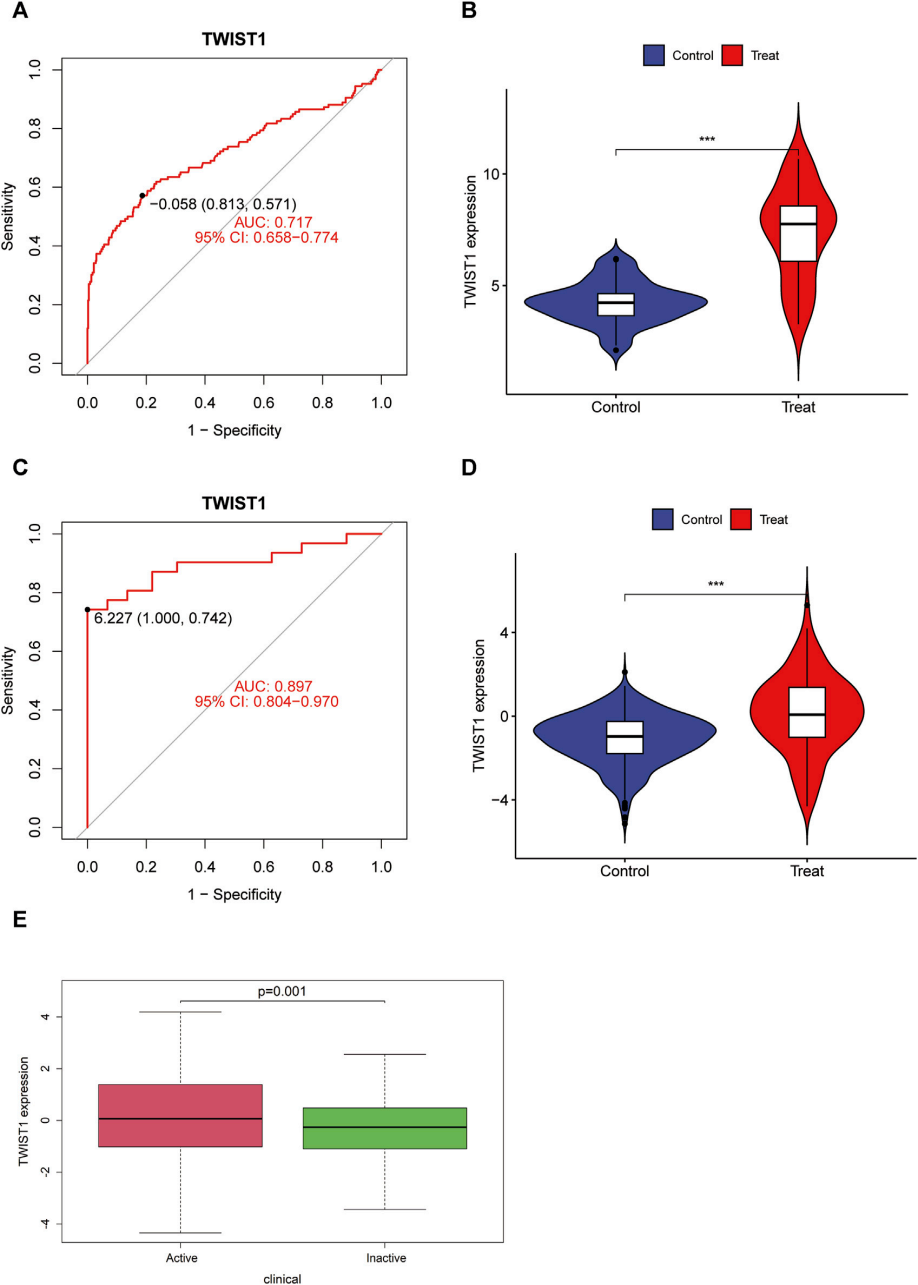
Receiver operating characteristic (ROC) curve, expression difference of TWIST1 gene and correlation between TWIST expression and disease activity in UC. **(A, B)** ROC curve and differential expression in GSE193677; **(C, D)** ROC curve and differential expression in GSE83687; **(E)** Boxplot of correlation between TWIST and UC clinical manifestation activity.

TWIST1 displayed significant expression differences in both GSE193677 and GSE83687 (Figures 4A, B). ROC curves were generated using data from GSE193677 and GSE83687, revealing TWIST1’s AUC to be 0.717 (95% confidence interval: 0.658–0.774) and 0.897 (95% confidence interval: 0.804–0.970) in GSE193677 and GSE83687, respectively (Figures 4C, D). In the GSE193677 dataset, a significant correlation was observed between high expression levels of TWIST1 and active clinical manifestations of UC (Figure 4E).

### 3.3 Identification of DEGs and enrichment analysis

**FIGURE 5:**
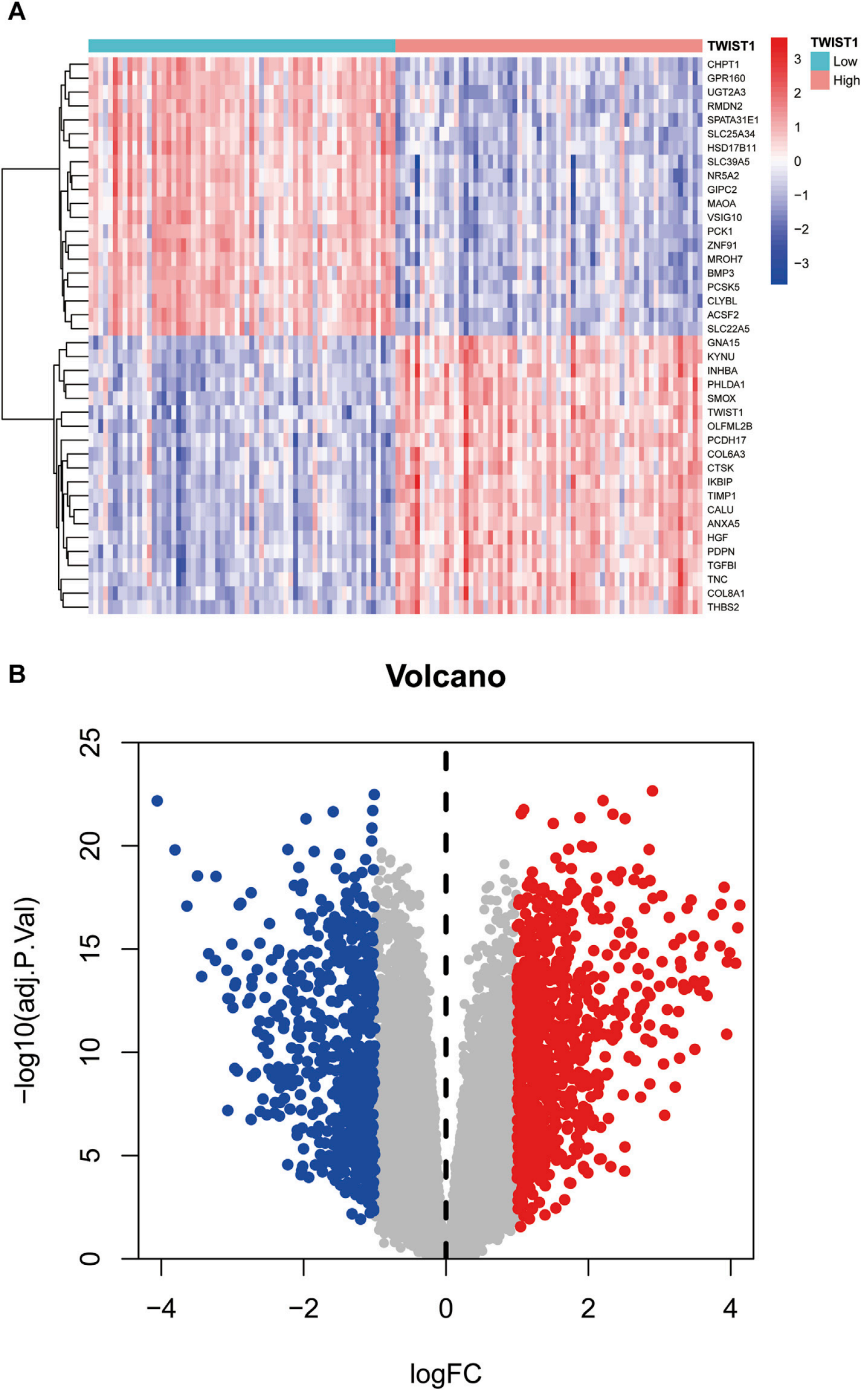
Heat map and volcano map based on the high and low expression groups of TWIST1 gene. **(A)** The heatmap of DEGs; **(B)** The volcano plots of DEGs. Red represented a high expression of DEG, while blue represented a low expression of DEG.

Within the UC sample of the GSE193677 dataset, a total of 1,518 DEGs were observed between the TWIST1 high expression and TWIST1 low expression groups, comprising 837 upregulated and 681 downregulated DEGs (Figure 5A, B).

**FIGURE 6:**
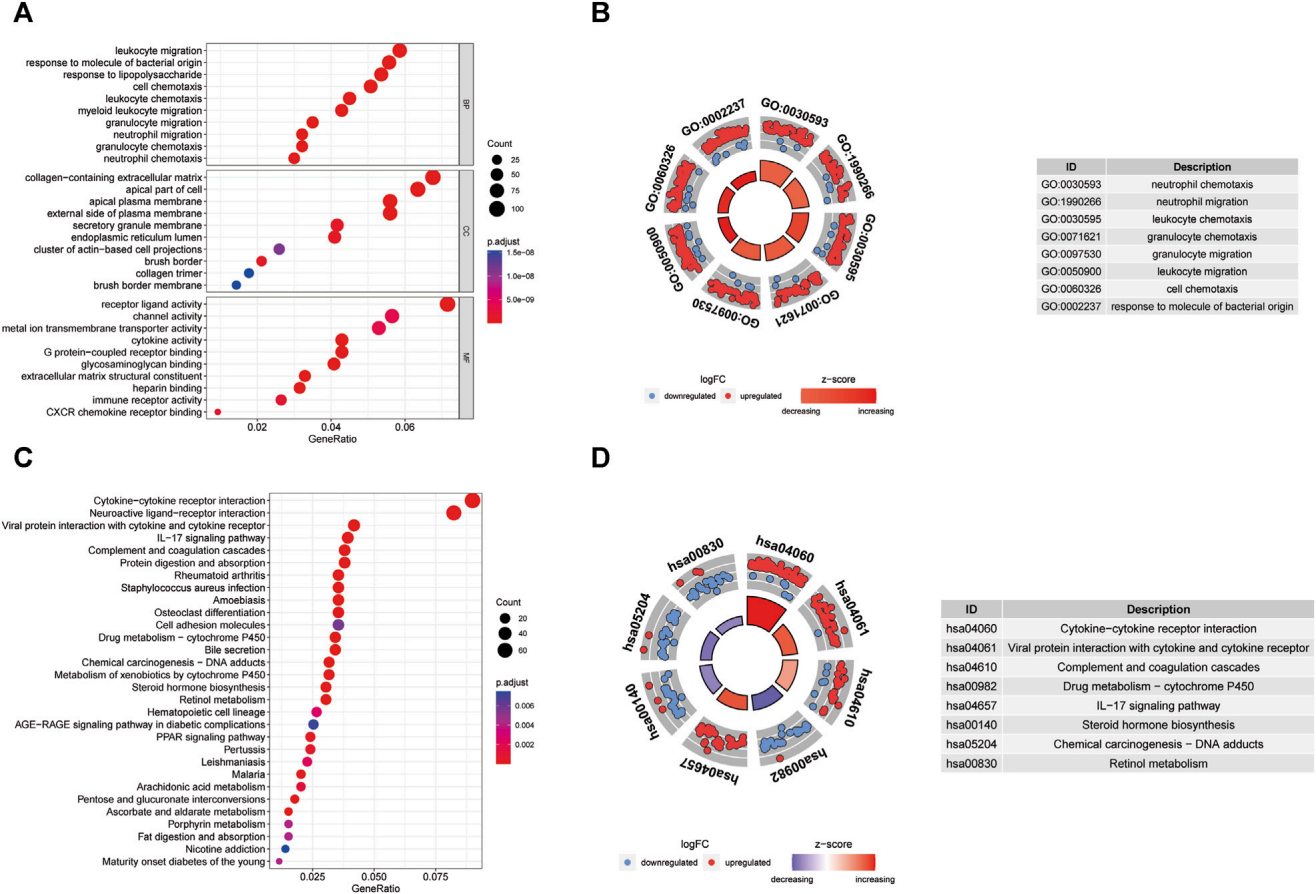
GO enrichment and KEGG analysis of DEGs. **(A)** Bubble plot of enriched GO terms. **(B)** Circos diagram of enriched GO terms. **(C)** Bubble plot of enriched KEGG terms. **(D)** Circos diagram of enriched GO terms. BP, biological process; CC, cellular component; MF, molecular function.

The GO enrichment analysis demonstrated that DEGs associated with BP were predominantly linked to activities such as leukocyte migration, humoral immune response, response to molecules of bacterial origin, response to lipopolysaccharide, and cell chemotaxis. For MF, DEGs were primarily engaged in receptor-ligand activity, channel activity, metal ion transmembrane transporter activity, and cytokine activity. Concerning CC, the distribution of DEGs was prominently observed in the collagen-containing extracellular matrix, apical part of the cell, and apical plasma membrane (Figures 6A, B). The KEGG pathway enrichment analysis unveiled the enrichment of DEGs in pathways including Cytokine-cytokine receptor interaction, Viral protein interaction with cytokine and cytokine receptor, as well as Complement and IL-17 signaling (Figures 6C, D).

**FIGURE 7:**
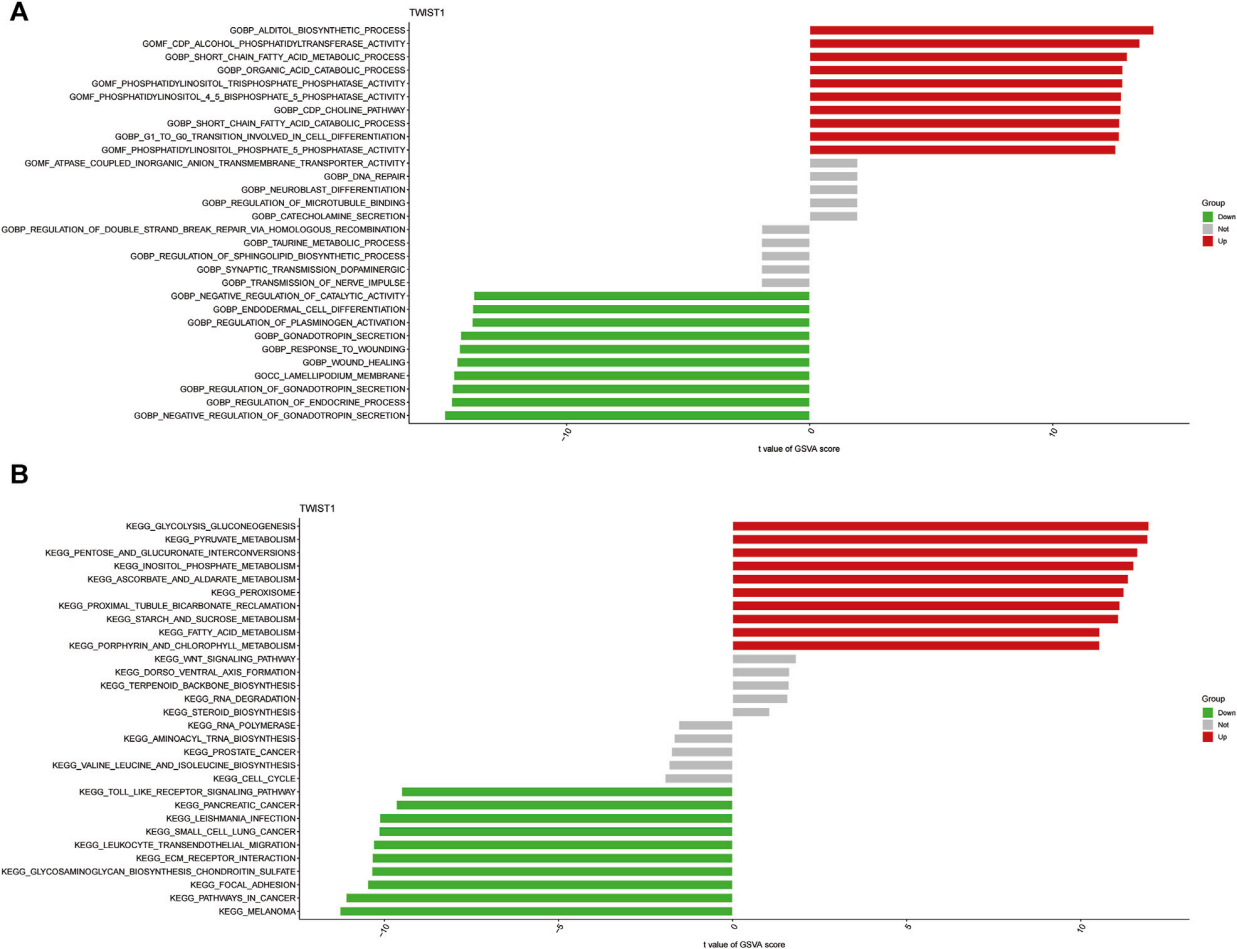
The analysis of GSVA indicates functional and pathway differences between high- and low-risk subgroups. **(A, B)** Variations in the terms of GO and KEGG pathways evaluated by GSVA between the TWIST1 high- and low-expression groups. The T values are shown using a linear model. The red column represents high enrichment in high expression groups, whereas the green column represents high enrichment in low expression groups.

GSVA was conducted to further explore the terms of GO and KEGG pathways between the TWIST1 high- and low-expression groups. Top 20 upregulated terms of GO and KEGG pathways were shown (Figures 7A, B). The results of the most significant enrichment of the two groups were shown in Table 2.

**TABLE 2:** The results of Gene Set Variation Analysis.

KEGG term	t	P-value	Sig
**KEGG_MELANOMA**	−11.2803	<0.001	Down
**KEGG_PATHWAYS_IN_CANCER**	−11.1048	<0.001	Down
**KEGG_FOCAL_ADHESION**	−10.4825	<0.001	Down
**KEGG_GLYCOSAMINOGLYCAN_BIOSYNTHESIS_CHONDROITIN_SULFATE**	−10.3610	<0.001	Down
**KEGG_ECM_RECEPTOR_INTERACTION**	−10.3503	<0.001	Down
**KEGG_PORPHYRIN_AND_CHLOROPHYLL_METABOLISM**	10.5423	<0.001	Up
**KEGG_FATTY_ACID_METABOLISM**	10.5470	<0.001	Up
**KEGG_STARCH_AND_SUCROSE_METABOLISM**	11.0846	<0.001	Up
**KEGG_PROXIMAL_TUBULE_BICARBONATE_RECLAMATION**	11.1238	<0.001	Up
**KEGG_PEROXISOME**	11.2410	<0.001	Up

GO term	t	P-value	Sig
**GOBP_NEGATIVE_REGULATION_OF_GONADOTROPIN_SECRETION**	−15.0196	<0.001	Down
**GOBP_REGULATION_OF_ENDOCRINE_PROCESS**	−14.7294	<0.001	Down
**GOBP_REGULATION_OF_GONADOTROPIN_SECRETION**	−14.6998	<0.001	Down
**GOCC_LAMELLIPODIUM_MEMBRANE**	−14.6394	<0.001	Down
**GOBP_WOUND_HEALING**	−14.5080	<0.001	Down
**GOMF_PHOSPHATIDYLINOSITOL_PHOSPHATE_5_PHOSPHATASE_ACTIVITY**	12.5935	<0.001	Up
**GOBP_G1_TO_G0_TRANSITION_INVOLVED_IN_CELL_DIFFERENTIATION**	12.7338	<0.001	Up
**GOBP_SHORT_CHAIN_FATTY_ACID_CATABOLIC_PROCESS**	12.7530	<0.001	Up
**GOBP_CDP_CHOLINE_PATHWAY**	12.8006	<0.001	Up
**GOMF_PHOSPHATIDYLINOSITOL_4_5_BISPHOSPHATE_5_PHOSPHATASE_ACTIVITY**	12.8234	<0.001	Up

### 3.4 CeRNA network construction of TWIST1 gene

**FIGURE 8:**
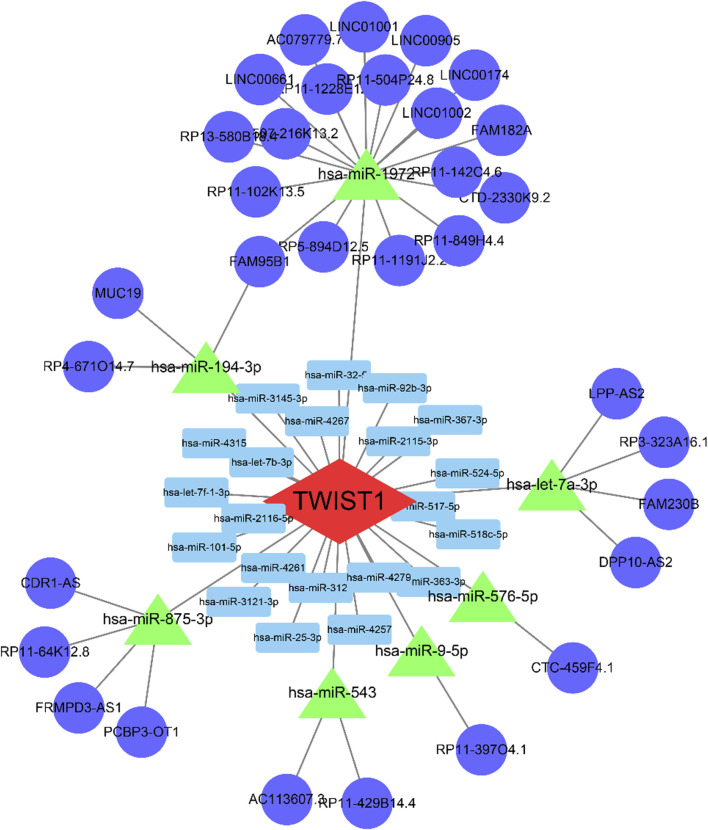
The ceRNA regulation network of 5 lncRNAs, 2 miRNAs, and 1 mRNA in patients. The blue circle indicates lncRNAs; the green rectangle indicates miRNAs; the red diamond indicates mRNA. lncRNA, long non-coding RNA; miRNA, microRNA.

The Supplementary Table S1 showcased the outcomes of mRNA-miRNA and lncRNA-miRNA analyses. We recognized 11 lncRNAs and 8 miRNAs, establishing their interactions through predictions and validations across databases like starBase, miRcode, Miranda, and TargetScan. The intricate interactions were graphically depicted using Cytoscape (Figure 8).

### 3.5 Infiltration of immune cells results

**FIGURE 9:**
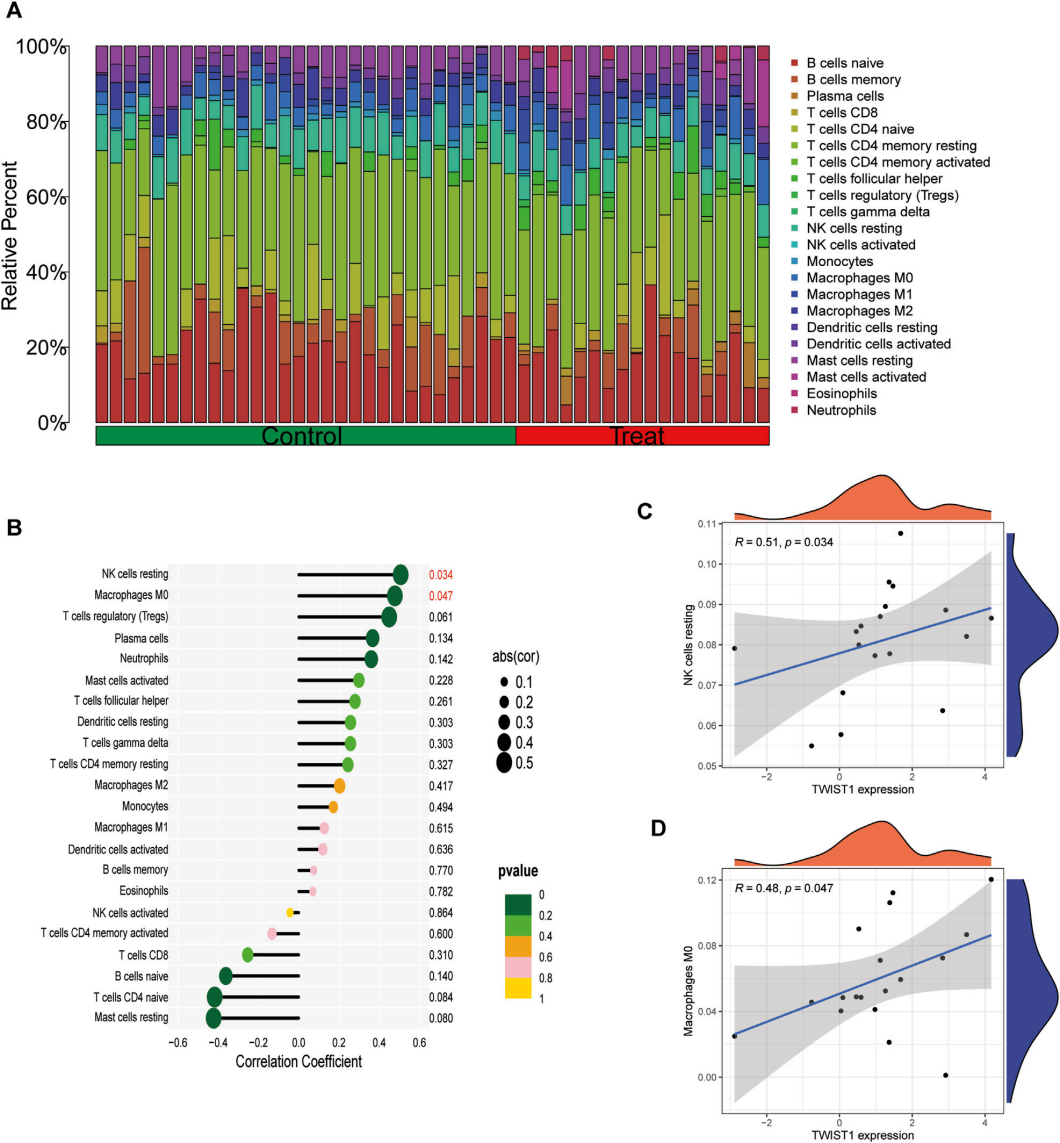
Analysis of TWIST1 and Immune Cell Infiltrates in different UC samples. **(A)** The relative percent of 22 kinds of immune cells in different lung samples. Differences in the levels of immune cells between the TWIST1 high- and low-expression groups in GSE193677. **(B)** Correlation analysis between TWIST1 and infiltrating immune cells in AS samples where red represented the positive correlation with a significant difference. **(C)** Scatter diagram indicating the correlation between TWIST1 expression and NK cells resting. **(D)** Scatter diagram indicating the correlation between TWIST1 expression and Macrophages M0 infiltration. Correlation analysis was assessed using Pearson correlation.

The infiltrated immune cells in different samples were analyzed using CIBERSORT and the overall relative abundances of 22 types of immune cells were shown (Figure 9A). The analysis results of infiltration degree of 22 immune cell showed that the infiltration of NK cells resting and Macrophages M0 was significantly different between TWIST1 high expression group and TWIST1 low expression group (Figure 9B). Further validation of the correlation study indicated that the expression level of S100A8 was correlated positively with NK cells resting and Macrophages M0 infiltration (r = 0.51, r = 0.48, all *p* < 0.05) (Figures 9C, D).

## 4 Discussion

UC, as one of the primary forms of IBD, is a chronic inflammatory intestinal disorder characterized by ulcers and inflammation within the intestinal tract (Kobayashi et al., 2020). The rise in Western dietary habits, improved socioeconomic status, enhanced sanitation, and advancements in vaccination have contributed to an increased incidence and prevalence of IBD in Asian countries (Park and Cheon, 2021). This surge is concomitant with a heightened occurrence of CAC. UC presents a significant clinical challenge, with its etiology and pathogenic mechanisms remaining largely elusive (De Souza and Fiocchi, 2016). This study aims to elucidate the potential role of the TWIST1 gene in UC through bioinformatics, machine learning, and functional enrichment analyses. Our findings offer invaluable insights into the molecular mechanisms of UC and underscore the potential of TWIST1 as a diagnostic and therapeutic target.

The Twist1 gene encodes a transcription factor encompassing a bHLH structural domain and is part of a protein family involved in organogenesis regulation (Thisse et al., 1988; Jan and January 1993; Kadesch, 1993). Recently, Twist1 has been established to play pivotal roles not only in the development of various organs and systems but also in cancer metastasis (Yang et al., 2004; Kwok et al., 2005; Puisieux et al., 2006; Cheng et al., 2008a; Cheng et al., 2008b; Li et al., 2009; Fu et al., 2011). Studies have indicated a pronounced elevation of TWIST1 protein in tissues from UC and UC-associated colorectal cancer, with the expression intensity being greater in the latter (Anonymous, 2023). Emerging perspectives suggest that histological inflammation and its severity are among the strongest drivers of CAC risk (Shah and Itzkowitz, 2022). The bHLH transcriptional repressor - TWIST1, acting as an antagonist for NF-κB-dependent cytokine expression, partakes in the modulation of inflammation-induced immunopathology (Niesner et al., 2008; Li et al., 2009). Additionally, Twist1 may also regulate Hand proteins (Hand 1 and 2) (Firulli and Conway, 2008) and Runx2 (Rice et al., 2000; Bialek et al., 2004). These downstream targets or interacting proteins of Twist1 are known to be involved in the development of various mesenchymal derivatives and multiple physiological functions.

Existing research has demonstrated the diagnostic value of elevated TWIST1 expression in UC through immunohistochemical techniques (Anonymous, 2023). Similarly, upon acquiring high-throughput sequencing data for UC, we categorized UC samples into high and low TWIST1 expression groups. Through machine learning, lasso regression, and ROC curve analysis, we validated the diagnostic significance of elevated TWIST1 expression in UC. It is widely recognized that immune homeostasis relies on immune cells and molecules, such as innate immune cells like NK cells and macrophages M0. In the UC mucosa, metabolic abnormalities in NK cells lead to secondary infections and increased cancer risk (Zaiatz Bittencourt et al., 2021), while macrophages M0 play a role in promoting mucosal immunity and inflammatory responses in UC (Peng et al., 2023). In our study, the upregulated expression of TWIST1 in UC also increased their impact on pro-immune and pro-inflammatory cells, providing immunological support for the role of TWIST1 in the progression of UC. We also conducted GO, KEGG, and GSVA analyses on the high and low TWIST1 expression groups to explore the pathways related to TWIST1 promoting UC development.

Upon identifying TWIST1 as a biomarker, we further predicted its associated miRNA and lncRNA using databases. Notably, a study in 2022 postulated a close association between miR-9-5p and the expression of NF-κB in UC tissues (Xu et al., 2022). NF-κB plays a pivotal role in regulating immune cells and cytokines (Mantovani et al., 2004; Wang et al., 2014), and animal studies have indicated that genetic defects in the negative regulators of the canonical NF-κB pathway heighten susceptibility to colonic inflammation (Zhang et al., 2006; Vereecke et al., 2014). This regulatory axis is crucial in the onset and progression of UC. Unfortunately, other regulatory axes identified in our study have been scarcely researched in the context of UC, warranting further exploration by the scientific community.

In this study, TWIST1 is highly correlated with UC, and previous research has also indicated a strong association between TWIST1 and UC-associated colorectal cancer (Kaz et al., 2010). Given that UC serves as a precancerous lesion for UC-associated colorectal cancer (Bopanna et al., 2017), our research provides valuable clues for investigating TWIST1 as a potential risk marker in the onset, development, and transformation of UC into UC-associated colorectal cancer. This offers research directions for the future prediction and treatment of UC and UC-associated colorectal cancer. However, it is important to acknowledge certain limitations in our study. Firstly, all the data analyzed through bioinformatics methods were directly obtained from US public databases, which may not fully represent the clinical scenarios in Asian populations. Secondly, the absence of sequencing data for CAC prevented us from concurrently evaluating the diagnostic significance of TWIST1 in both UC and CAC.

## Data Availability

The data presented in this study are deposited in the GitHub repository, accessible at: https://github.com/xia-wanqiu/single_gene_analysis_for_UC.git. The sample data, GSE193677 and GSE83687, presented in this study are sourced from public databases.

## Abbreviations

AUC, Area under the curve; IBD, Inflammatory bowel diseases; GEO, Gene Expression Omnibus; DEGs, Differentially expressed genes; ceRNA, Competing endogenous RNA; FDR, False discovery rate; GO, Gene ontology analysis; BP, Biological processes; CC, Cellular components; MF, Molecular functions; KEGG, Kyoto Encyclopedia of genes and Genomes; GSVA, Gene Set Variation Analysis; ROC, Receiver Operating Characteristic; lncRNA, Long non-coding RNA; NK, Natural killing; FC Fold Change; CAC, colitis-associated colorectal cancer; SVM-RFE, Support Vector Machine with Recursive Feature Elimination; LASSO, Least Absolute Shrinkage and Selection Operator; S100P, S100 Calcium Binding Protein P; GPR15, G protein-coupled receptor 15; TWIST1, Twist Family BHLH Transcription Factor 1; RND1, Rho Family GTPase 1; bHLH, basic helix-loop-helix; UC, Ulcerative colitis; .
