# Supplementary material for: Elucidating the role of TWIST1 in ulcerative colitis: a comprehensive bioinformatics and machine learning approach
Source: Front Genet. 2024 Mar 6;15:1296570. doi: 10.3389/fgene.2024.1296570 (PMC10952112; doi:10.3389/fgene.2024.1296570)
Supplement: Supplementary file 1 [file Table1.docx]

| **Node** | **Type** |
| --- | --- |
| TWIST1 | mRNA |
| hsa-miR-1972 | miRNA |
| hsa-miR-875-3p | miRNA |
| hsa-miR-576-5p | miRNA |
| hsa-let-7a-3p | miRNA |
| hsa-miR-194-3p | miRNA |
| hsa-miR-543 | miRNA |
| hsa-miR-9-5p | miRNA |
| LINC00661 | lncRNA |
| RP11-64K12.8 | lncRNA |
| CTC-459F4.1 | lncRNA |
| DPP10-AS2 | lncRNA |
| CH507-216K13.2 | lncRNA |
| LINC00905 | lncRNA |
| RP4-671O14.7 | lncRNA |
| CDR1-AS | lncRNA |
| RP11-1228E12.1 | lncRNA |
| FAM230B | lncRNA |
| FAM182A | lncRNA |
| RP11-504P24.8 | lncRNA |
| LINC00174 | lncRNA |
| RP13-580B18.4 | lncRNA |
| FAM95B1 | lncRNA |
| RP11-429B14.4 | lncRNA |
| RP11-102K13.5 | lncRNA |
| LINC01001 | lncRNA |
| CTD-2330K9.2 | lncRNA |
| RP11-142C4.6 | lncRNA |
| LINC01002 | lncRNA |
| RP3-323A16.1 | lncRNA |
| RP11-849H4.4 | lncRNA |
| RP11-397O4.1 | lncRNA |
| RP5-894D12.5 | lncRNA |
| AC113607.3 | lncRNA |
| LPP-AS2 | lncRNA |
| MUC19 | lncRNA |
| RP11-1191J2.2 | lncRNA |
| PCBP3-OT1 | lncRNA |
| FRMPD3-AS1 | lncRNA |
| AC079779.7 | lncRNA |

| **miRNA** | **lncRNA** | **Interaction** |
| --- | --- | --- |
| TWIST1 | hsa-miR-3125 | mRNA |
| TWIST1 | hsa-let-7b-3p | mRNA |
| TWIST1 | hsa-miR-4315 | mRNA |
| TWIST1 | hsa-let-7f-1-3p | mRNA |
| TWIST1 | hsa-miR-367-3p | mRNA |
| TWIST1 | hsa-miR-9-5p | mRNA |
| TWIST1 | hsa-miR-4279 | mRNA |
| TWIST1 | hsa-miR-4267 | mRNA |
| TWIST1 | hsa-miR-2115-3p | mRNA |
| TWIST1 | hsa-miR-25-3p | mRNA |
| TWIST1 | hsa-miR-32-5p | mRNA |
| TWIST1 | hsa-miR-518c-5p | mRNA |
| TWIST1 | hsa-miR-194-3p | mRNA |
| TWIST1 | hsa-miR-3121-3p | mRNA |
| TWIST1 | hsa-miR-1972 | mRNA |
| TWIST1 | hsa-miR-576-5p | mRNA |
| TWIST1 | hsa-miR-2116-5p | mRNA |
| TWIST1 | hsa-miR-92b-3p | mRNA |
| TWIST1 | hsa-miR-524-5p | mRNA |
| TWIST1 | hsa-miR-875-3p | mRNA |
| TWIST1 | hsa-miR-3145-3p | mRNA |
| TWIST1 | hsa-miR-517-5p | mRNA |
| TWIST1 | hsa-miR-101-5p | mRNA |
| TWIST1 | hsa-let-7a-3p | mRNA |
| TWIST1 | hsa-miR-543 | mRNA |
| TWIST1 | hsa-miR-363-3p | mRNA |
| TWIST1 | hsa-miR-4257 | mRNA |
| TWIST1 | hsa-miR-4261 | mRNA |
| FAM230B | hsa-let-7a-3p | lncRNA |
| RP5-894D12.5 | hsa-miR-1972 | lncRNA |
| RP11-397O4.1 | hsa-miR-9-5p | lncRNA |
| FAM95B1 | hsa-miR-194-3p | lncRNA |
| DPP10-AS2 | hsa-let-7a-3p | lncRNA |
| LINC01002 | hsa-miR-1972 | lncRNA |
| RP11-102K13.5 | hsa-miR-1972 | lncRNA |
| RP4-671O14.7 | hsa-miR-194-3p | lncRNA |
| MUC19 | hsa-miR-194-3p | lncRNA |
| FAM182A | hsa-miR-1972 | lncRNA |
| RP11-504P24.8 | hsa-miR-1972 | lncRNA |
| CTC-459F4.1 | hsa-miR-576-5p | lncRNA |
| RP13-580B18.4 | hsa-miR-1972 | lncRNA |
| FRMPD3-AS1 | hsa-miR-875-3p | lncRNA |
| RP11-849H4.4 | hsa-miR-1972 | lncRNA |
| LINC01001 | hsa-miR-1972 | lncRNA |
| RP11-142C4.6 | hsa-miR-1972 | lncRNA |
| AC113607.3 | hsa-miR-543 | lncRNA |
| LINC00174 | hsa-miR-1972 | lncRNA |
| RP11-64K12.8 | hsa-miR-875-3p | lncRNA |
| RP3-323A16.1 | hsa-let-7a-3p | lncRNA |
| LINC00905 | hsa-miR-1972 | lncRNA |
| PCBP3-OT1 | hsa-miR-875-3p | lncRNA |
| CDR1-AS | hsa-miR-875-3p | lncRNA |
| FAM95B1 | hsa-miR-1972 | lncRNA |
| CTD-2330K9.2 | hsa-miR-1972 | lncRNA |
| RP11-1228E12.1 | hsa-miR-1972 | lncRNA |
| AC079779.7 | hsa-miR-1972 | lncRNA |
| RP11-1191J2.2 | hsa-miR-1972 | lncRNA |
| RP11-429B14.4 | hsa-miR-543 | lncRNA |
| LPP-AS2 | hsa-let-7a-3p | lncRNA |
| LINC00661 | hsa-miR-1972 | lncRNA |
| CH507-216K13.2 | hsa-miR-1972 | lncRNA |
